# Identify risk factors for perioperative outcomes in Intracorporeal Urinary Diversion and Extracorporeal Urinary Diversion with Robotic Cystectomy

**DOI:** 10.1590/S1677-5538.IBJU.2023.0477

**Published:** 2024-03-18

**Authors:** Hangcheng Fu, Laura Davis, Venkat Ramakrishnan, Thomas Barefoot, Colleen Sholtes, Lifan Liang, Mohammed Said, Jamie Messer

**Affiliations:** 1 University of Louisville School of Medicine Department of Urology Louisville KY United States Department of Urology, University of Louisville School of Medicine, Louisville, KY, United States;; 2 University Hospital Cleveland Medical Center Case Western Urology Institute Cleveland OH United States Case Western Urology Institute, University Hospital Cleveland Medical Center, Cleveland OH, United States;; 3 Brigham and Women's Hospital Division of Urology Boston MA USA Division of Urology, Brigham and Women's Hospital, Boston. MA, USA;; 4 Cleveland Clinic Akron General Urology & Pelvic Health Center Department of Urology OH United States Department of Urology, Urology & Pelvic Health Center, Cleveland Clinic Akron General, OH, United States;; 5 University of Louisville School of Medicine Department of Medicine Louisville KY United States Department of Medicine, University of Louisville School of Medicine, Louisville, KY, United States

**Keywords:** Urinary Bladder Neoplasms, Robotic Surgical Procedures, Urinary Diversion

## Abstract

**Introduction:**

The increasing adoption of robotic-assisted cystectomy with intracorporeal urinary diversion (ICUD), despite its complexity, prompts a detailed comparison with extracorporeal urinary diversion (ECUD). Our study at a single institution investigates perioperative outcomes and identifies risk factors impacting the success of these surgical approaches.

**Methods:**

In this retrospective analysis, 174 patients who underwent robotic-assisted cystectomy at the University of Louisville from June 2016 to August 2021 were reviewed. The cohort was divided into two groups based on the urinary diversion method: 30 patients underwent ECUD and 144 underwent ICUD. Data on demographics, complication rates, length of hospital stay, and readmission rates were meticulously collected and analyzed.

**Results:**

Operative times were comparable between the ICUD and ECUD groups. However, the ICUD group had a significantly lower intraoperative transfusion rate (0.5 vs. 1.0, p=0.02) and shorter hospital stay (7.8 vs. 12.3 days, p<0.001). Factors such as male sex, smoking history, diabetes mellitus, intravesical therapy, higher ASA, and ACCI scores were associated with increased Clavien-Dindo Grade 3 or higher complications. Age over 70 was the sole factor linked to a higher 90-day readmission rate, with no specific characteristics influencing the 30-day rate.

**Conclusion:**

Robotic cystectomy with ICUD results in shorter hospitalizations and lower intraoperative transfusion rates compared to ECUD, without differences in operative time, high-grade postoperative complications, or readmission rates. These findings can inform clinical decision-making, highlighting ICUD as a potentially more favorable option in appropriate settings.

## INTRODUCTION

Bladder cancer is the fourth most common cancer in men in the United States and as such, creates a substantial financial burden at both the individual and the national level. Radical cystectomy with urinary diversion remains the standard surgical approach for non-metastatic muscle-invasive bladder cancer (MIBC) as well as for certain cases of high-risk non-muscle invasive bladder cancer (NMIBC) (1). On the other hand, simple cystectomy was used in certain refractory diseases of radiation cystitis, neurogenic bladder, interstitial cystitis, or incontinence in addition to urinary diversion. Initially, open cystectomy with extracorporeal diversion was the preferred surgical approach. Since the advent of robotic-assisted procedures, however, studies have set out to compare the safety, efficacy, and perioperative and oncologic outcomes of open vs. robotic-assisted laparoscopic cystectomy(2–7). Recently, multiple RCT trials compared open cystectomy vs. robotic cystectomy with intracorporeal urinary diversion were published (7–10). Different urinary diversions have been reported to be performed in either intracorporeal or extracorporeal fashion (11). A topic that has been less closely examined is the comparison of intracorporeal urinary diversion (ICUD) vs. extracorporeal urinary diversion (ECUD) following robotic-assisted laparoscopic cystectomy with different types of urinary diversion.

Despite several studies comparing perioperative outcomes between ICUD and ECUD, most are large database studies with unclear results. We hypothesized that patients with intracorporeal urinary diversion (ICUD) may potentially have a faster bowel recovery time. To address this issue, we present our study from a single institution and aim to use the perioperative outcomes including perioperative blood loss, transfusion, operative times, postoperative complications rates, and 30-day and 90-day readmission rates to identify the demographic feature or operative techniques that may influence the outcome.

## MATERIALS AND METHODS

### Study Design

This retrospective study, approved by the Human Subjects Office/Institutional Review Board (IRB) at the University of Louisville (IRB number 20.0406), involved 174 patients who underwent robotic-assisted cystectomy at the University of Louisville Hospital between May 2016 and July 2021.

Inclusion criteria were as follows: patients who underwent planned robotic total cystectomy with urinary diversion, with or without concurrent procedures. Patients were excluded if they underwent partial cystectomy, had anesthetic contraindications to robotic surgery, or lacked demographic information or post-operative follow-up data.

Baseline demographics included the patient's age, gender, BMI, smoking status, bladder cancer status, history of diabetes mellitus, type of urinary diversion (ileal conduit, neobladder, or Indiana pouch), method of urinary diversion (intracorporeal or extracorporeal), ECOG status, ASA, ACCI score, neoadjuvant chemotherapy status, intravesical treatment status, and history of pelvic radiation.

Patients were divided into two groups based on the method of urinary diversion employed: the ECUD group and the ICUD group. The study classified 30 patients into the ECUD group and 144 into the ICUD group. The primary outcome measured was complications of Clavien-Dindo Grade III or above. Secondary outcomes focused on 30-day and 90-day readmission rates. All the patients in our study were initiated with ERAS protocol and were encouraged to start a liquid diet and ambulation post-operative day one. No narcotic was regularly prescribed postoperatively. The detailed protocol was described in the supplementary documentary 2 in APPENDIX.

The study also considered other perioperative outcomes, including operative time, postoperative ileus, day of initiating per oral intake (PO), length of stay, urine leak, and bowel leak. Postoperative ileus was defined as postoperative vomiting paired with radiographic evidence of ileus that necessitated nasogastric tube (NG tube) placement. The day of initiating PO was defined as the day when the patient started a solid diet post-surgery. For the study's integrity, patients lacking 90-day follow-up information were excluded from the research.

### Procedure

All surgeries involved in this study were performed by the same surgeon using the Da Vinci Robot Xi system and encompassed both radical and simple robotic cystectomy procedures. An extended bilateral pelvic lymph node dissection was carried out on all patients undergoing radical cystectomy. This dissection included perivesical, external iliac, common iliac, obturator, and presacral lymph nodes.

While the operations adhered to a standard template, variations were made as necessary for specific cases. Given that the ileal conduit was the most frequently used method of urinary diversion in this cohort, a detailed template of the robotic cystectomy and both intracorporeal and extracorporeal ileal conduit creation is provided in supplementary document 1 in APPENDIX for reference. Different techniques were reported to reconstruct neobladder, and we used a Studer/Wiklund technique in our orthotopic neobladder reconstruction (12, 13). The robotic intracorporeal continent cutaneous urinary diversion (Indiana pouch) was performed in a similar fashion as previously reported (14).

### Statistical Analysis

We used the chi-square test to compare the association between category variables. Student's t-test was used to analyze the association between continuous variables in patients’ demographics. All P values were two-tailed with differences considered significant at values of P<0.05. Statistical analysis was performed with MedCalc software (version 18.2.1; MedCalc, Mariakerke, Belgium).

## RESULTS

### Patients’ baseline demographics between the ECUD group and the ICUD group

In total, 174 patients were enrolled in this study, with 30 assigned to the ECUD group and 144 to the ICUD group. As detailed in z, no significant differences were found between the two subgroups in terms of age, gender, BMI, preoperative diagnosis, smoking status, diabetes mellitus status, neoadjuvant chemotherapy, intravesical treatment, baseline ECOG scores, ASA scores, and ACCI scores.

The choice of urinary diversion method correlated with the type of urinary diversion. Specifically, 66.7% (16/24) of patients with an Indiana pouch underwent extracorporeal surgery, while all 42 patients with a neobladder underwent the procedure intracorporeally. Furthermore, a larger proportion of patients in the extracorporeal subgroup had received pelvic radiotherapy before the surgery compared to the ICUD group (23.3% vs. 7.6%, p = 0.010).

### Comparison of the perioperative outcomes between ECUD and ICUD subgroups

As detailed in Table-1, there was no statistically significant difference in operative time between the ECUD and ICUD groups. The median operative time for the ECUD group was 303.5 minutes, compared to 287.0 minutes for the ICUD group. However, patients who underwent ECUD required more transfusions on average than those in the ICUD group (1.0 vs. 0.5, p=0.020).

**Table 1:** Patient demographics and perioperative outcomes of the UofL cohort between ECUD and ICUD.

		All patients (%)	ECUD (%)	ICUD (%)	*P*
**Gender** 1					0.114
	Female	49 (28.2)	12 (6.9)	37 (21.3)	
	Male	125 (71.8)	18 (10.3)	107 (61.5)	
**Age** 2		66 (58-74)	62 (57-75)	66 (58.5-73.5)	0.710
**BMI** 2		29.3 (24.5-34.5)	27.5 (22.8-31.8)	29.6 (25.1-35.1)	0.552
**Diversion type** 1					<0.001
	Ileal Conduit	103 (59.2)	13 (7.5)	90 (51.7)	
	Indiana Pouch	24 (13.8)	16 (9.2)	8 (4.6)	
	Neobladder	42 (24.1)	0	42 (24.1)	
	Others	5 (2.9)	1 (0.6)	4 (2.3)	
**Preoperative diagnosis** 1					0.670
	Bladder cancer	134 (77.0)	24 (13.8)	110 (63.2)	
	Others	40 (23.0)	6 (3.4)	34 (19.5)	
	Cystectomy types				0.565
	Simple cystectomy	34 (19.5)	7 (4.0)	27 (13.2)	
	Radical cystectomy	140 (80.5)	23 (13.2)	117 (67.2)	
**Smoking status** 1					0.196
	Never	38 (21.8)	10 (5.7)	28 (16.1)	
	Former smoker	78 (44.8)	10 (5.7)	68 (39.1)	
	Current smoker	58 (33.3)	10 (5.7)	48 (27.6)	
**Diabetes Mellitus** 1					0.523
	No	119 (68.4)	22 (12.6)	97 (55.7)	
	Yes	55 (31.6)	8 (4.6)	47 (27.0)	
**Neoadjuvant Chemotherapy** 1					0.102
	No	117 (67.2)	24 (13.8)	93 (53.4)	
	Yes	57 (32.8)	6 (3.4)	51 (29.3)	
**Intravesical treatment** 1					0.189
	No	140 (80.5)	25 (14.4)	115 (66.1)	
	Yes	34 (19.5)	5 (2.9)	29 (16.7)	
**Pelvic Radiotherapy** 1					0.010
	No	156 (89.7)	23 (13.2)	133 (76.4)	
	Yes	18 (10.3)	7 (4.0)	11 (6.3)	
**ECOG** 1					0.726
	0	115 (66.1)	19 (10.9)	96 (55.2)	
	≥1	59 (33.9)	11(6.3)	48 (27.6)	
**ASA** 1					0.718
	≤3	153 (87.9)	25 (14.4)	128 (73.6)	
	≥4	21 (12.1)	5 (2.9)	16 (9.2)	
**ACCI** 1					0.207
	≤6	129 (74.1)	25 (14.4)	104 (59.8)	
	≥7	45 (25.9)	5 (2.9)	40 (23.0)	
Operative time (minutes)					0.743
	Mean±SD	303.2±81.4	307.5±79.3	294.0±76.3	
	Median (25%-75%)	302 (241-349)	303 (248-358)	287 (242-341)	
Intraoperative Transfusion (unit)					0.020
	Mean±SD	0.7±1.7	1.0±1.6	0.5±1.2	
	Median (25%-75%)	0 (0-0)	0 (0-2)	0 (0-0)	
Postoperative Transfusion (unit)					<0.001
	Mean±SD	0.6±1.2	1.2±1.7	0.4±1.0	
	Median (25%-75%)	0 (0-0)	0 (0-2)	0 (0-0)	
Length of Stay (days)					<0.001
	Mean±SD	9.5±8.0	12.3±8.7	7.8±5.3	
	Median (25%-75%)	7 (5-9)	9 (7-16)	7 (5-9)	
Days initiating PO					0.029
	Mean±SD	4.9 ± 4.3	6.1±3.0	4.7±2.7	
	Median (25%-75%)	5 (3-7)	5.5 (4-7)	4(3-7)	
Days to Flatus					0.002
	Mean±SD	3.5 ± 1.8	4.4±3.0	3.3 ± 1.5	
	Median (25%-75%)	3 (2-4)	4 (3-5)	3 (2-4)	
Days to bowel movement					0.003
	Mean±SD	4.2±2.0	5.2±2.8	4.0±1.7	
	Median (25%-75%)	4(3-5)	5 (3-6)	4(3-5)	
Clavien Dinno Grade 3 or above complication					0.077
	Yes	52 (29.9)	13 (9.8)	105 (60.3)	
	No	122 (70.1)	17 (7.5)	39 (22.4)	

ASA = American Society of Anesthesiology score; ACCI = Age-adjusted Charlson Comorbidity Index scores; ECUD = extracorporeal urinary diversion; ECOG = Eastern Cooperative Oncology Group performance status; ICUD = intracorporeal urinary diversion; SD = standard deviation;

1 chi-square test was used for categorized variables

2 t independent test was used for continuous variables, Median (25%-75%)

Additionally, patients in the ICUD group initiated a diet earlier than those in the ECUD subgroup (median day 4 vs. 5.5, p=0.029). They also had an earlier recovery for both flatus (median day 4 vs. 3) and bowel movement (median day 5 vs. 4). The average length of hospital stay was longer for patients in the ECUD group than in the ICUD group (12.3 vs. 7.8 days, p<0.001), with a median stay of 9 days for ECUD patients compared to 7 days for those in the ICUD group.

### The association between Clavien-Dino Grade 3 above complications and patient characteristics

In this study, 29.8% of patients experienced Clavien-Dindo Grade 3 or higher complications. In the ECUD subgroup, 13 out of 40 patients (43.3%) were diagnosed with Clavien-Dindo Grade 3 or above complications, while 39 out of 144 patients (27%) in the ICUD group experienced the same. However, no statistical difference was found regarding the risk of high-grade complications between these two groups.

Upon further analysis to identify potential risk factors for high-grade complications, it was found that the male gender exhibited a higher risk compared to the female, with an odds ratio (OR) of 2.330 (p=0.041). Patients who underwent other types of urinary diversion (including percutaneous ureterostomy, ureterosigmoidostomy, and colon conduit) were more likely to be associated with high-grade complications compared to those who had an ileal conduit, with an OR of 11.259 (p=0.033). Other factors associated with Clavien-Dindo Grade 3 or higher complications were a history of smoking, previous intravesical treatment, high ASA score, and high ACCI score, as shown in Table-2. A separate subgroup analysis was performed regarding the pelvic radiation risk in different cystectomy types (simple vs. radical) patient populations. In this subgroup analysis, pelvic radiotherapy was significantly correlated to higher Clavien-Dinno 3 risk with OR 5.4 (1.1-26.9, p =0.039) in the radical cystectomy subgroup while pelvic radiation is not statistically significantly associated with higher Clavien-Dinno 3 complication risk (p=0.141) in the simple cystectomy subgroup.

**Table 2:** Patient characteristics and association with Clavien-Dino Grade 3 above complications.

Characteristics		OR (95% CI)	P
**Univariable**	
	Gender (Male vs. Female^1^)	2.330 (1.035-5.249)	0.041
	Age (≥70 vs. <70 1)	1.044 (0.539-2.025)	0.898
**BMI**			
	30-40 vs. <30 1	1.080 (0.540-2.159)	0.827
	≥40 vs. <30 1	1.692 (0.547-5.230)	0.360
Method of Diversion (ICUD vs. ECUD^1^)		0.485 (0.216-1.092)	0.080
**Diversion type**			
	Indiana Pouch vs. Ileal conduit^1^	2.010 (0.799-5.058)	0.138
	Neobladder vs. Ileal conduit^1^	0.998 (0.441-2.259)	0.997
	Others 2 vs. Ileal conduit 1	11.259 (1.205-105.225)	**0.033**
Preoperative diagnosis (Others vs. Bladder cancer 1)		0.511 (0.217-1.202)	0.124
Radical cystectomy vs. Simple cystectomy 1		0.916 (0.409-2.054)	0.832
**Smoking status**			
	Former smoker vs. Never smoker 1	2.768 (1.082-7.075)	**0.033**
	Current smoker vs. Never smoker 1	1.545 (0.563-4.237)	0.398
Diabetes Mellitus (Yes vs. No 1)		2.516 (1.274-4.970)	**0.008**
Neoadjuvant Chemotherapy (Yes vs. No 1)		0.513 (0.244-1.078)	0.078
Intravesical treatment (Yes vs. No 1)		2.198 (1.013-4.767)	**0.046**
Pelvic Radiation therapy (Yes vs. No 1)		2.036 (0.754-5.497)	0.160
ECOG (≥1 vs. 0 1)		0.816 (0.407-1.638)	0.568
ASA (≥4 vs. ≤3^1^)		3.005 (1.187-7.601)	**0.020**
ACCI (≥7 vs. ≤6^1^)		2.126 (1.043-4.330)	**0.037**
**Multivariable**			
Gender (Male vs. Female^1^)		1.685 (0.717-3.959)	0.230
**Diversion type**			
	Indiana Pouch vs. Ileal conduit 1	1.435 (0.448-4.594)	0.542
	Neobladder vs. Ileal conduit 1	0.853 (0.340-2.138)	0.734
	Others 2 vs. Ileal conduit 1	2.695 (0.168-43.215)	0.483
**Smoking status**			
	Former smoker vs. Never smoker 1	3.354 (1.094-10.285)	**0.034**
	Current smoker vs. Never smoker 1	2.354 (0.733-7.558)	0.150
Diabetes Mellitus (Yes vs. No 1)		2.327 (1.111-4.858)	**0.025**
Intravesical treatment (Yes vs. No 1)		1.893 (0.776-4.616)	0.160
ASA (≥4 vs. ≤3 1)		1.719 (0.552-5.355)	0.345
ACCI (≥7 vs. ≤6 1)		1.585 (0.687-3.657)	0.280

ASA = American Society of Anesthesiology score; ACCI = Age-adjusted Charlson Comorbidity Index scores; ECUD = extracorporeal urinary diversion; ECOG = Eastern Cooperative Oncology Group performance status; ICUD = intracorporeal urinary diversion; SD = standard deviation;

1 calculated as a reference

2 Including percutaneous ureterostomy, ureterosigmoidostomy, colon conduit, etc

Then, we performed multivariable analysis with logistic regression including all the factors that were previously statistically significant. Interestingly, only smoking history and diabetes were found to be associated with high Clavien-Dinno 3 complication risk (p =0.034 and p = 0.025, respectively).

### The association between 30-day and 90-day readmission and patient characteristics

Of the 174 patients in the study, 46 (26.4%) required readmission within 30 days, and 59 (33.9%) were readmitted within 90 days. In the ECUD group, 26.6% of patients were readmitted within 30 days, comparable to the 26.3% in the ICUD group. Regarding 90-day readmission, 40% of patients in the ECUD group were readmitted, compared to 32.6% in the ICUD group.

An association analysis of 30-day readmission risk with patient characteristics is presented in Table-S1. No significant correlations were found between 30-day readmission and factors like patient gender, age, BMI, method of diversion, type of diversion, cystectomy types, preoperative diagnosis, smoking status, diabetes mellitus, neoadjuvant chemotherapy, intravesical treatment, pelvic radiation therapy, ECOG score, ASA score, and ACCI score. A similar analysis was conducted for 90-day readmission risk. It was found that patients aged less than 70 years had a lower risk of 90-day readmission, with an odds ratio of 0.490 (p=0.037), suggesting that younger age is a protective factor against 90-day readmission.

## DISCUSSION

The debate surrounding the advantages of ICUD versus ECUD has been ongoing since the emergence of robotic surgery. However, years later, there is still a scarcity of data and conflicting results regarding the perioperative outcomes of these two surgical procedures. In this study, we aimed to scrutinize the perioperative outcomes in patients who underwent ICUD and ECUD after robotic-assisted cystectomy. All operations were performed by a single surgeon at our institution, helping to provide further insight into this complex issue.

In our research, we found that ICUD was the preferred procedure, outnumbering ECUD. This outcome was not surprising considering that patient randomization was not part of our study design. Despite this, preoperative patient demographics, including average BMI, gender, and median age at the time of the procedure, showed no significant difference between the two groups. This suggests that specific patient characteristics did not notably influence the choice of one surgical method over the other. However, patients with a history of pelvic radiotherapy and those who underwent Indiana Pouch creation were more likely to have ECUD, perhaps due to the increased technical difficulties associated with performing ICUD in these groups. These findings from our single-institution study align with previous research investigating this topic at an international level (15–17). Mazzone et al. reported that ICUD in highly comorbid patients has a lower risk of postoperative complications rate compared to ECUD (18). However, in this study, we found no significant difference between the two procedures. Both ICUD and ECUD groups displayed comparable rates of high-grade Clavien-Dindo complications (defined as Clavien-Dindo Grade 3 or higher), as well as 30 or 90-day readmission rates.

Conclusions regarding perioperative transfusion rates have varied in previous studies, with some indicating no difference between the two methods of urinary diversion, while others suggest a reduced need for intraoperative transfusion in patients undergoing ICUD (15,16, 19, 20). Our research also found a decreased requirement for intraoperative transfusion in the ICUD group, with rates at 0.5 compared to 1.0 for the ECUD group. This finding bears significant relevance considering its implications on a patient's disease course. Increased perioperative transfusions following radical cystectomy have been associated with a higher risk of both cancer recurrence and mortality (21,22).

A principal concern related to the use of ICUD is the potential increase in operative time, attributed to the technical challenges posed by a fully intracorporeal procedure (23–26). Prolonged operative time becomes especially problematic for patients undergoing any robotic surgery, as the requisite use of CO2 for insufflation may be challenging for certain patients with pre-existing cardiopulmonary comorbidities. Moreover, extended usage may lead to acidosis. Given these considerations, it's noteworthy that we found no substantial difference in operative time between ICUD (294.0 min) and ECUD (301.5 min). This aligns with prior studies demonstrating that as surgeons gain more experience performing robotic procedures, operative times reduce, potentially rivaling those of open procedures (27–29).

The significant operational costs of a robotic surgical system have often been cited as a drawback to adopting robotic surgical approaches, with the direct and indirect costs of a robotic procedure estimated to be around $4250 (30). Although the surgery was commonly performed in the Da Vinci platform, ICUD was also reported to be done in different systems (31). However, we observed that patients undergoing ICUD initiated oral intake sooner, consequently leading to a shorter hospital stay. Thus, a portion of these costs may be counterbalanced in patients undergoing ICUD, as reduced length of stay can decrease direct costs for both patients and healthcare systems (29, 30). Our findings demonstrate a shorter hospital stay in the ICUD group, with an average of 7.8 days versus 12.3 days in the ECUD group. The potential cost savings implicit in this difference are significant for both the hospital system and patients. Given sufficient patient volume, these savings could even offset the costs of purchasing and maintaining robotic systems over time.

Our study, being retrospective, has inherent selection biases. Additionally, the distribution of patients undergoing ICUD compared to ECUD was uneven, owing to the non-randomized nature of this investigation. It's also important to note that the consistency in operative time between ICUD and ECUD observed in this study may not be universally applicable, given that the procedures were performed by a single surgeon experienced in robotic techniques. This study mainly focuses on the perioperative outcome instead of long-term complications, late complications are not uncommon in this population including ureteral ileal stenosis, chronic kidney disease, and urinary tract infection (32). Future studies can be designed to focus on the long-term complication outcomes. Also, both simple cystectomy and radical cystectomy were included which can potentially increase confounding factors of the study given different disease nature and lack of lymph nodes dissection in simple cystectomy subgroup. While one could argue that our results have limited generalizability, considering they're based on outcomes from a single institution and surgeon, our approach also bypasses the decreased specificity often resulting from larger database studies, which is a strength of our work. A multicenter retrospective study with a propensity score match could potentially decrease the selection bias (33). Further randomized studies are indeed necessary to clarify these findings and fill the existing data gap on this topic. Additionally, an analysis of cost versus savings between the two surgical approaches could shed more light on the cost benefits to both patients and hospital systems resulting from a reduced length of stay.

Our results indicate that with a proficient robotic surgeon, the operative time – often considered a limiting factor in executing this procedure – doesn't differ significantly between ICUD and ECUD. Additionally, we observed that the hospital stay was substantially shortened, and the transfusion rate improved in the ICUD group compared to the ECUD group. Despite these differences, we found no significant variance between the two groups in terms of postoperative complication rates or readmission rates. These findings may suggest that the intracorporeal approach to urinary diversion can provide certain advantages without increasing postoperative complications or readmission rates, particularly when performed by a surgeon well-versed in robotic procedures.

## COMPLIANCE WITH ETHICAL STANDARDS

The Human Subjects Office/Institutional Review Board (IRB) reviewed our study. This retrospective study, approved by the Human Subjects Office/Institutional Review Board (IRB) at the University of Louisville (IRB number 20.0406),

## APPENDIX:

**Supplementary Table 1:** Patient characteristics and association with 30-days and 90-days readmission rate

Characteristics		30-days readmission		90-days readmission	
		OR (95% CI)	P	OR (95% CI)	P
Gender (Male vs. Female 1)		0.993 (0.469-2.099)	0.986	0.952 (0.475-1.909)	0.891
Age (<70vs. ≥70 1)		0.663 (0.326-1.347)	0.256	0.490 (0.250-0.960)	0.037
**BMI**					
	30-40 vs. <30^1^	1.643 (0.804-3.361)	0.173	1.713 (0.879-3.337)	0.113
	≥40 vs. <30^1^	1.800 (0.551-5.871)	0.329	1.692 (0.547-5.229)	0.361
Method of Diversion (ICUD vs. ECUD 1)		1.272 (0.383-4.228)	0.693	0.726 (0.323-1.632)	0.439
Diversion type					
	Indiana Pouch vs. Ileal conduit^1^	1.355 (0.503-3.654)	0.548	1.822 (0.727-4.565)	0.200
	Neobladder vs. Ileal conduit^1^	1.475 (0.664-3.277)	0.339	1.735 (0.818-3.677)	0.150
	Others vs. Ileal conduit^1^	2.194 (0.346-13.909)	0.404	3.827 (0.607-24.101)	0.152
Preoperative diagnosis (Others vs. Bladder cancer 1)		1.313 (0.571-3.022)	0.521	1.471 (0.675-3.206)	0.331
Smoking status					
	Former smoker vs. Never smoker^1^	1.968 (0.760-5.093)	0.162	1.534 (0.664-3.540)	0.316
	Current smoker vs. Never smoker^1^	1.545 (0.563-4.237)	0.398	1.104 (0.451-2.703)	0.827
Diabetes Mellitus (Yes vs. No^1^)		1.581 (0.781-3.199)	0.202	1.477 (0.760-2.871)	0.249
Neoadjuvant Chemotherapy (Yes vs. No^1^)		0.990 (0.483-2.032)	0.979	0.855 (0.435-1.681)	0.650
Intravesical treatment (Yes vs. No^1^)		0.826 (0.344-1.982)	0.668	0.773 (0.342-1.748)	0.537
Pelvic Radiation therapy (Yes vs. No^1^)		1.450 (0.510-4.118)	0.485	1.647 (0.613-4.424)	0.322
ECOG (0 vs. ≥1^1^)		0.608 (0.287-1.287)	0.194	0.550 (0.274-1.103)	0.092
ASA (≥4 vs. ≤3^1^)		1.862 (0.717-4.835)	0.201	1.929 (0.768-4.847)	0.162
ACCI (≥7 vs. ≤6^1^)		1.580 (0.754-3.312)	0.225	1.431 (0.709-2.888)	0.317

ECUD = extracorporeal urinary diversion; ICUD = intracorporeal urinary diversion; SD = standard deviation

1 calculated as a reference

## Supplementary Document 1 Technique Description of Robotic Cystectomy with Intracorporeal Ileal Conduit Creation and Extracorporeal Ileal Conduit Creation in Male

**Robotic cystectomy with Intracorporeal ileal conduit creation (Male)**

1. the patient was placed in low lithotomy position with all pressure points padded.
2. A paramedian left upper quadrant 12 mm trocar was placed by modified Hasson Technique. Pneumoperitoneum was then established. Four additional 8 mm robotic trocars were placed for triangulation to the bladder/cecum. A left lateral 5 mm trocar placed for assistant. The Davinci XI robot was then docked in a typical sterile fashion.
3. The sigmoid colon was reflected out of the pelvis and the left ureter was identified in the retroperitoneum. This was dissected to the vesical hiatus and clipped distally with a 10 mm hemo-lok clip, and proximally with a 10 mm hemo-lok with a Vicryl tag. The right ureter was handled in similar manner after identification in the retroperitoneum.
4. We then made an incision in the pouch of Douglas and developed the space between the rectum and the bladder/prostate.
5. Incision was made lateral to the right medial umbilical ligament and the space of Retzius was developed. This was extended to the right vas which was used as a handle. The right superior vesical artery was clipped with a 10 mm Hemo-lok and divided with a Davinci vessel sealer.
6. The posterior bladder pedicle was divided with a vessel sealer to the apical prostate. The left space of Retzius was developed in a similar manner. The left side bladder pedicle handled in a similar manner.
7. Once this was accomplished the medial and median umbilical ligaments were divided and the space of Retzius was completely developed.
8. The bladder was retracted out of the pelvis and the puboprostatic ligaments were identified and exposed. These were divided and the dorsal vein divided with cautery and vessel sealer. The apical urethra was divided sharply and the urethra was closed with a 3-0 Vicryl to prevent spillage.
9. The terminal ileum was identified and divided in 20 cm proximal to the Ileocecal valve, and again 15 cm proximal to this incision for the future ileal conduit using a Echelon 60 mm stapler.
10. The mesenteric pedicle was developed with the vessel sealer.
11. A side to side ileo-ileostomy was then performed using the Echelon 60 mm stapler.
12. The enterotomy was closed in 2 layers with 3-0 PDS.
13. Bilateral ureteral anastomosis carried out in a two layer fashion using 3-0 Vicryl to anastomose adventitia to the seromuscular layer of the pouch.
14. Mucosal anastomosis after wide spatulation of the ureter with 4-0 Monocryl.
15. Prior to closure of the ureter 7F bander ureteral stents placed and secured into the conduit using 3-0 Chromic through the stent. Stents brought through the stomal end.
16. The Pelvis was then irrigated and suctioned out
17. A 19 round Blake drain was placed in the pelvis through a 8 mm trocar
18. Stoma matured in the previously marked RLQ space with 2-0 Vicryl in a standard Brooke Fashion.
19. The Hasson trocar was then extended and the specimen extracted.
20. Fascia was closed using interrupted figure of eight 1 PDS sutures. The Hasson trocar was closed using a figure of eight 0 PDS.
21. Skin closed with 4-0 Monocryl.
22. The port incisions were closed with 4-0 Monocryl. The patient had a 19 round Blake drains placed through the left side 8 mm trocar.

**Extracorporeal ileal conduit creation (Male)**

*Following the step 8 from Robotic cystectomy with Intracorporeal ileal conduit creation (Male)*

1. We then identified the patient's cecum and terminal ileum. A segment of terminal ileum approximately 15 cm from the ileocecal valve was marked using a 3-0 Vicryl stitch.
2. At this portion of the procedure, we then converted to an open procedure along the midline. On the proximal side of the ileum we isolated a 15 cm segment for use as the ileal conduit using the previous mark.
3. Bilateral ureteral anastomosis carried out in a two layer fashion using 3-0 Vicryl to anastomose adventitia to the seromuscular layer of the pouch.
4. Mucosal anastomosis after wide spatulation of the ureter with 4-0 Monocryl.
5. Prior to closure of the ureter 7F bander ureteral stents placed and secured into the conduit using 3-0 Chromic through the stent. Stents brought through the stomal end.
6. The Pelvis was then irrigated and suctioned out.
7. The stoma matured in the LUQ in a typical brook fashion.
8. A 19 round Blake drain was placed in the pelvis through the 8 mm trocar.
9. The Hasson trocar was closed using a figure of eight 0 Vicryl. Skin closed with 4-0 Monocryl. The port incisions were closed with 4-0 Monocryl.
10. The midline was closed with interrupted 0 PDS figure of eight sutures, and skin in the midline closed with staples.

## Supplementary Document 2 Postoperatively ERAS protocol in robotic cystectomy patients. Robotic cystectomy with Intracorporeal ileal conduit creation (Male)

Following an unremarkable intraoperative event, our patients adhered to an Enhanced Recovery After Surgery (ERAS) protocol, emphasizing a faster recovery with reduced complications. Postoperatively, all patients were transferred to a specialized urology floor. The recovery process began on the night of the surgery, with patients being encouraged to sit in a chair and consume hard candy along with small sips of clear liquid. . Of note, nasogastric tube was not routinely placed. Pain management was a critical component of our protocol. We employed a combination of IV Toradol, oral ibuprofen, and epidural anesthetics, deliberately avoiding oral or IV narcotics to mitigate potential side effects and enhance recovery. Dietary progression was carefully monitored, with patients starting on a clear liquid diet from the first postoperative day. Advancement to a soft solid diet was contingent on evidence of returning bowel function, indicated by flatus or bowel movement. This gradual dietary transition played a significant role in patient comfort and bowel recovery. Additionally, we placed a strong emphasis on early mobilization. Patients were encouraged to ambulate aggressively starting from postoperative day 1. This early physical activity is a cornerstone of ERAS and has been shown to significantly contribute to reducing postoperative complications and hastening recovery. All patients were started DVT prophylaxis before intubation and immediately after surgery unless concerned for bleeding postoperatively. We don't routinely continue antibiotics postoperatively.
